# The American Pharmacopœia

**Published:** 1882-07

**Authors:** 


The American Homœopathic Pharmacopœia. Compiled and published by Bœricke & Tafel: New York and Philadelphia. Price, $3.50. pp. 524. 1882.

The preface says: "For many years the want of a practical, complete and reliable homœopathic pharmacopœia has been felt in this country."

After a careful examination of this work, we feel we can safely affirm that Messrs. Bœricke & Tafel have fully supplied this want. The book meets with the hearty approval of Prof. T. F. Allen, who should certainly be considered an authority upon therapeuties and pharmacology.

